# Modeling Spectral Properties in Stationary Processes of Varying Dimensions with Applications to Brain Local Field Potential Signals

**DOI:** 10.3390/e22121375

**Published:** 2020-12-05

**Authors:** Raanju R. Sundararajan, Ron Frostig, Hernando Ombao

**Affiliations:** 1 Department of Statistical Science, Southern Methodist University, Dallas, TX 75275, USA; 2 School of Biological Sciences, University of California Irvine, Irvine, CA 92697, USA; rfrostig@uci.edu; 3 Statistics Program, King Abdullah University of Science and Technology, Thuwal 23955, Saudi Arabia; hernando.ombao@kaust.edu.sa

**Keywords:** multivariate time series, nonstationary, spectral matrix, local field potential, 62M10, 62M15

## Abstract

In some applications, it is important to compare the stochastic properties of two multivariate time series that have unequal dimensions. A new method is proposed to compare the spread of spectral information in two multivariate stationary processes with different dimensions. To measure discrepancies, a frequency specific spectral ratio (FS-ratio) statistic is proposed and its asymptotic properties are derived. The FS-ratio is blind to the dimension of the stationary process and captures the proportion of spectral power in various frequency bands. Here we develop a technique to automatically identify frequency bands that carry significant spectral power. We apply our method to track changes in the complexity of a 32-channel local field potential (LFP) signal from a rat following an experimentally induced stroke. At every epoch (a distinct time segment from the duration of the experiment), the nonstationary LFP signal is decomposed into stationary and nonstationary latent sources and the complexity is analyzed through these latent stationary sources and their dimensions that can change across epochs. The analysis indicates that spectral information in the Beta frequency band (12–30 Hertz) demonstrated the greatest change in structure and complexity due to the stroke.

## 1. Introduction

Numerous applications require comparing two multivariate time series of unequal dimensions. Neuroscience experiments result in a stationary or nonstationary multivariate signal from different epochs (distinct non-overlapping successive time segments of the duration of the experiment). A popular approach to modeling such data decomposes the observed signal at every epoch into useful latent sources that can be stationary or nonstationary. These latent sources are lower dimensional time series obtained by linear transforms of the components of the observed multivariate series and they aim to capture important statistical properties of the observed series. At these epochs, dimension reduction techniques such as principal component analysis (PCA), factor modeling, independent component analysis (ICA), stationary subspace analysis (SSA) are often applied to extract useful lower-dimensional latent sources. Artificially setting the dimension of these latent sources to be the same across the epochs results in loss of important information since these changes could be indicative of useful brain processes such as learning (Fiecas and Ombao [1]). Indeed brain processes evolve across the entire recording period (Fiecas and Ombao [1], Ombao et al. [2]) leading to changes in the dimension of the latent sources across epochs. Moreover, the evolution of the dimension can itself serve as a feature in understanding how the brain function evolves during an experiment. As another example in neuroscience, the aim in functional connectivity is to model dependence between different brain regions at various epochs in an experiment; Cribben et al. [3], Cribben et al. [4], Cribben and Yu [5], Zhu and Cribben [6]. To mitigate the problem of high-dimensionality arising due to signal from densely voxelated cortical surface, parcellation leads to disjoint regions of interest (ROI) of the brain and signal summaries are obtained in each of these regions. Dependence measures between these ROIs are then computed using their respective signal summaries. In the above pursuit of region-wise comparison of the brain, it is natural to encounter the problem of comparing multivariate processes, say from two different regions that have unequal dimensions. In Wang et al. [7] the problem of modeling effective connectivity in high-dimensional cortical surface signal is pursued wherein a factor analysis is carried out on each ROI and vector autoregressive (VAR) models are used to jointly model the latent factors. Here again, one can potentially end up with unequal number of optimal latent factors from different ROIs thereby making the comparisons challenging.

**Figure 1:**
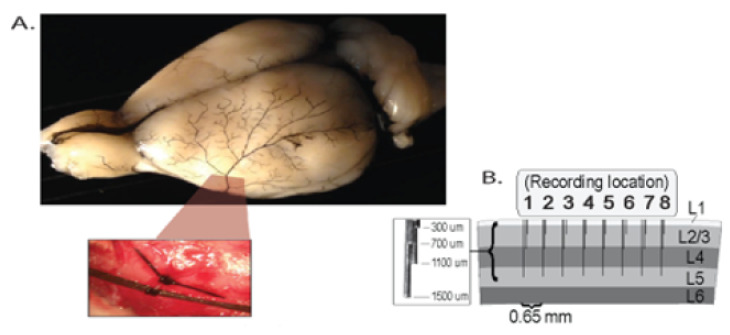
(**A**) Visual representation of the 32 microelectrodes on the rat’s cortex from which the local field potential (LFP) signal is recorded. (**B**) The distance between microelectrodes is 0.65 mm and the total distance between microelectrode 1 and microelectrode 8 is 3.9 mm.

The application that motivates our methodology is the analysis of local field potentials (LFP) in an experiment that simulates ischemic stroke in humans (Data source: Stroke experiment conducted in the lab of co-author (Ron Frostig) at his Neurobiology lab; http://frostiglab.bio.uci.edu/Home.html). The dataset comprises of 600 epochs worth of LFP recordings (each epoch is 1 s long) from 32 microelectrodes implanted in a rat’s cortex. Figure 1 below depicts the rat’s cortex and the locations of the 32 sensors implanted on the cortical surface from which the LFP signal is recorded. This 32-dimensional signal is our observed time series. A stroke is induced midway through the experiment (epoch 300) by clamping the medial cerebral artery. The goal is to develop a method that tracks changes in the complexity of signals following the stroke. From the observed LFP signal, useful lower-dimensional sources are extracted at each epoch and we shall characterize complexity in LFP through these useful latent sources and their varying dimensions across epochs.

Motivated by such applications, we propose a new method to compare spectral information in different multivariate stationary processes of varying dimensions. More specifically, the aim is to capture the amount of spectral information in various frequency bands in different stationary processes of unequal dimensions. There are already many methods and models that discuss evolution of spectral information but the key contribution of this paper is in modeling evolution of the spectrum while allowing dimension to also evolve over time. We introduce a frequency-specific spectral ratio, which we call the FS-ratio, statistic that measures the proportion of spectral power in various frequency bands. FS-ratio can be used to (i). identify frequency bands where there is significant discrepancies between pre and post stroke epochs, (ii). identify frequency bands that account for most variation within pre (and post) stroke epochs and (iii). identify the frequency bands that are consistent (vs inconsistent) across all the 600 epochs. One of the key features of this statistic is that it is blind to the dimension of the multivariate stationary process and can be used to compare successive epochs with possibly different dimensions in the stationary sources. Thus, the proposed FS-ratio is very useful in (a). discriminating between the pre and post stroke onset and (b). tracking changes over the entire course of the experiment while allowing for varying dimensions. In Section 2 we develop our FS-ratio statistic and derive its asymptotic properties. We return to the LFP dataset in Section 3 and discuss the usefulness of the proposed ratio statistic in discriminating between pre and post stroke onset. Section 4 concludes. Finally, we evaluate the performance of the proposed FS-ratio statistic through several simulation examples and the results are provided in Appendix A.

## 2. Methodology

In this section, we first describe our FS-ratio statistic and the method to analyze the evolution of spectral information in stationary processes with varying dimensions. Using the FS-Ratio statistic, a technique to locate the frequency bands carrying significant spectral power is discussed in Section 2.1.1. The theoretical properties of the proposed statistic along with the required assumptions are discussed in Section 2.1.2.

### 2.1. The FS-Ratio Statistic

Let $X_{t}$ be a $d_{1}$-variate time series and $Y_{t}$ be a $d_{2}$-variate time series where $d_{1}\neq d_{2}$ and t=1,2,…,T. The spectral matrices of the two zero-mean multivariate stationary series are given by $f_{X}\left(\omega \right)\in C^{d_{1}\times d_{1}}$ and $f_{Y}\left(\omega \right)\in C^{d_{2}\times d_{2}}$ for ω∈(−π,π). Here (−π,π) represents the normalized frequency range used to take care of aliases in the frequency components outside this range. This range (−π,π) is sometimes referred to as angular frequency scale with frequency 2π being called the Nyquist or folding frequency. With the discrete Fourier transforms of $X_{t}$ and $Y_{t}$ expressed as $J_{X,T}\left(\omega \right)=\frac{1}{\sqrt{2\pi T}}X_{t}e^{-it\omega }$ and $J_{Y,T}\left(\omega \right)\;=\;\frac{1}{\sqrt{2\pi T}}Y_{t}e^{-it\omega }$, respectively, the periodogram matrices $I_{X,T}\left(\omega \right)\in C^{d_{1}\times d_{1}}$ and $I_{Y,T}\left(\omega \right)\in C^{d_{2}\times d_{2}}$ of the two series are obtained by

(1) $$I_{X,T}\left(\omega \right)=J_{X,T}\left(\omega \right)J_{X,T}{\left(\omega \right)}^{*}\;\;\mathrm{and}\;\;I_{Y,T}\left(\omega \right)=J_{Y,T}\left(\omega \right)J_{Y,T}{\left(\omega \right)}^{*},$$

where $J_{X,T}{\left(\omega \right)}^{*}$ denotes the conjugate transpose. The estimated spectral matrices, for ω∈(−π,π), are given by

(2) $${\hat{f}}_{X}\left(\omega \right)=\frac{1}{T}\sum_{j=-\lfloor \frac{T}{2}\rfloor +1}^{\left\lfloor \frac{T}{2}\right\rfloor }\;\;K_{h}\left(\omega -\omega_{j}\right)\;I_{X,T}\left(\omega_{j}\right)\;\;\mathrm{and}\;\;{\hat{f}}_{Y}\left(\omega \right)=\frac{1}{T}\sum_{j=-\lfloor \frac{T}{2}\rfloor +1}^{\left\lfloor \frac{T}{2}\right\rfloor }\;\;K_{h}\left(\omega -\omega_{j}\right)\;I_{Y,T}\left(\omega_{j}\right),$$

where $\omega_{j}=\frac{2\pi }{T}j$ and $K_{h}\left(\cdot \right)=\frac{1}{h}K\left(\frac{\cdot }{h}\right)$ where K(·) is a nonnegative symmetric kernel function and *h* denotes the bandwidth. Assumptions on the kernel and bandwidth to ensure uniform consistency in ω∈(−π,π) of the estimated spectral matrices are listed in Section 2.1.2.

The aim of this work is to compare the two spectral matrices $f_{X}\left(\omega \right)$ and $f_{Y}\left(\omega \right)$ over a specific frequency range (a,b) for some 0<a<b<π. The challenge here, however, is that the dimensions of the processes $X_{t}$ and $Y_{t}$ are unequal and hence their spectral matrices have varying dimensions. We thus focus on the spread or distribution of spectral power in each of these stationary processes across different frequency ranges. More precisely, for the $d_{1}$-variate series $X_{t}$ we define the frequency-specific spectral (FS-ratio) parameter as

(3) $$R_{X,a,b}\;=\;\frac{\;r_{X,a,b}}{r_{X,0,\pi }}\;=\;\frac{\int_{a}^{b}||vec\left(f_{X}\left(\omega \right)\right){\left|\right|}_{2}^{2}d\omega }{\int_{0}^{\pi }||vec\left(f_{X}\left(\omega \right)\right){\left|\right|}_{2}^{2}d\omega }$$

for some frequency band (a,b)⊂(0,π) where vec(·) denotes vectorization of a matrix into a single column vector and $\left|\right|\cdot {\left|\right|}_{2}^{2}$ is the squared Euclidean norm. Observe that $R_{X,a,b}\in \left(0,1\right)$ can be viewed as a measure that captures the proportion of spectral power found in the frequency range (a,b). Similarly using the spectral matrix $f_{Y}\left(\omega \right)$, $R_{Y,a,b}\in \left(0,1\right)$ can be defined for the $d_{2}$-variate series $Y_{t}$. Comparisons can now be made between the parameters $R_{X,a,b}$ and $R_{Y,a,b}$ to understand the amount of spectral power in the frequency range (a,b) for the two multivariate series with unequal dimensions.

The data analogue of the FS-ratio parameter in (3) is then given by the FS-ratio statistic:

(4) $${\hat{R}}_{X,a,b}\;=\;\frac{\;{\hat{r}}_{X,a,b}}{{\hat{r}}_{X,0,\pi }}\;=\;\frac{\int_{a}^{b}||vec\left({\hat{f}}_{X}\left(\omega \right)\right){\left|\right|}_{2}^{2}d\omega }{\int_{0}^{\pi }||vec\left({\hat{f}}_{X}\left(\omega \right)\right){\left|\right|}_{2}^{2}d\omega }$$

for some 0<a<b<π. Similarly, the data analogue ${\hat{R}}_{Y,a,b}$ can be obtained for the $d_{2}$-variate series $Y_{t}$. The asymptotic properties of the quantities ${\hat{r}}_{X,a,b}$ and ${\hat{R}}_{X,a,b}$ are discussed in Section 2.1.2. In neuroscience applications such as the one in Section 3, pre-defined frequency bands such as Theta, Alpha, Beta and Gamma are often used to understand the distribution of spectral power across these frequency bands. As opposed to using pre specified frequency bands, in Section 2.1.1 below we provide a data-driven technique to locate the various frequency ranges (a,b) that carry significant spectral power.

#### 2.1.1. Finding Frequency Bands of Interest

In this section we describe our technique that uses the FS-Ratio statistic to find the frequency bands of interest. More precisely, we aim to locate the intervals (a,b) used in (3) and (4) wherein the multivariate time series has significant proportions of spectral power.

**Figure 2:**
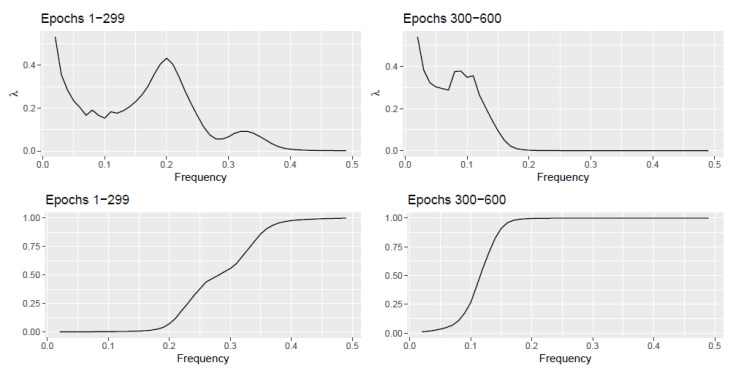
**Example 1** (**Top**) Plot of average scan statistic ${\hat{\lambda }}_{X,a_{j}}$ for epochs i<300 (**left**) and i≥300 (**right**) at a discretized sequence of frequency points $0<a_{1}=0.01<a_{2}=0.02<\ldots <a_{49}=0.49<0.5$ (Δ=0.01). Here (0,0.5) corresponds to the interval (0,π). (**Bottom**) Plot of the average of the statistic ${\hat{R}}_{X,0,a_{j}}$ at the same frequency points.

Let $X_{t}$ be a $d_{1}$-variate zero-mean second order stationary time series with its $d_{1}\times d_{1}$ spectral matrix given by $f_{X}\left(\omega \right)$. With the FS-Ratio parameter defined in (3), we consider the scan parameter

(5) $$\lambda_{X,a}=1-\frac{R_{X,0,a-\Delta }}{R_{X,0,a}}=1-\frac{\int_{0}^{a-\Delta }||vec\left(f_{X}\left(\omega \right)\right){\left|\right|}_{2}^{2}d\omega }{\int_{0}^{a}||vec\left(f_{X}\left(\omega \right)\right){\left|\right|}_{2}^{2}d\omega }$$

for a small Δ>0 and 0<a<π. For the data analogue of the parameter above we consider a discretized sequence of frequency points $0<a_{1}<a_{2}<\ldots <a_{Q}<\pi$ and evaluate the scan statistic as

(6) $${\hat{\lambda }}_{X,a_{j}}=1-\frac{{\hat{R}}_{X,0,a_{j}}}{{\hat{R}}_{X,0,a_{j+1}}}$$

for j=1,2,…,Q−1. A plot of the scan statistic ${\hat{\lambda }}_{X,a_{j}}$ across the various frequency points $a_{j}$ will indicate the frequency ranges over which the spectral matrix of $X_{t}$ has significant proportions of spectral power. Typically, one notices upward bumps in these plots over frequency ranges that carry significant spectral power; see Example 1 below and the top panel of Figure 2. Similarly for the $d_{2}$-variate series $Y_{t}$ one can define $\lambda_{Y,a}$, find the estimated version ${\hat{\lambda }}_{Y,a_{j}}$ and obtain the plot of it across the various frequency points $a_{j}$. Comparisons can then be made between the series $X_{t}$ and $Y_{t}$ using these plots. The choice for Δ in (5) and number of points *Q* in (6) depends on the application under consideration. Certain applications demand attention to spectral power in very small frequency ranges and certain others might not. A multiscale approach can also be used where one obtains plots of the scan statistic ${\hat{\lambda }}_{X,a_{j}}$ across frequency points $a_{j}$ for a sequence of Δ values. Visual inspection of these plots will help detect frequency ranges wherein the upward bumps are consistent across most values of Δ. If we let the interval (0,0.5) correspond to the interval (0,π), our simulation study and real data analysis indicate a choice of Δ=0.01 and Q=49 as reasonable.

We next provide a simple illustration of the scan statistic ${\hat{\lambda }}_{X,a}$ through the following simulation example and show how it is useful in detecting important frequency ranges. The simulation scheme in this illustration is designed to mimic the real data situation in Section 3. There the entire duration of the neuroscience experiment is divided into non-overlapping successive time segments (a total of *N* epochs). The multivariate stationary processes of interest in these *N* epochs tend to have different dimensions and we attempt to mimic that scenario.

**Example** **1.** *We consider N stationary processes, $X_{1,t},X_{2,t},\ldots ,X_{N,t}$, with the series $X_{i,t}$ given by*

(7) $$X_{i,t}=\left\{\begin{array}{cc} V_{i,t}^{\left(1\right)} & if\;i<\frac{N}{2}\\ V_{i,t}^{\left(2\right)} & if\;i\geq \frac{N}{2} \end{array}\right.$$

*where i=1,2,…,N=600 epochs, t=1,2,…,T=1000. Here $V_{i,t}^{\left(1\right)}\in R^{3}$ and its components are given by $v_{0,t+k-1}+v_{1,t+k-1}$ for k=1,2,3 and $v_{0,t}$ follows a AR(2) with (−0.8,−0.7) and $v_{1,t}$ follows a AR(2) with (0.25,−0.75). The components of $V_{i,t}^{\left(2\right)}\in R^{2}$ are given by $v_{2,t+k-1}$ for k=1,2 and $v_{2,t}$ follows a AR(2) with (1.25,−0.75).*

We consider a discretized set of frequency points $\left\{a_{1},a_{2},\ldots ,a_{Q}\right\}$ of the interval (0,π). At each point $a_{j}$ we evaluate the average of the scan statistic ${\hat{\lambda }}_{X,a_{j}}$ over epochs 1-299 and the average of the scan statistic over epochs 300–600. More precisely at each frequency point $a_{j}$ and at each epoch, we obtain ${\hat{\lambda }}_{X,a_{j}}$ and compute averages of these quantities over the respective epochs. In the top panel of Figure 2 we plot this average scan statistic. For epochs 1–299, $V_{i,t}^{\left(1\right)}$ from (7) is a combination of two AR(2) processes with spectral density peaks at roughly 0.22 and 0.33. The top left plot in Figure 2 witnesses the scan statistic exhibiting bumps around those frequencies. Similarly for epochs 300–600, $V_{i,t}^{\left(2\right)}$ from (7) is generated from an AR(2) process with peak at roughly 0.12. The top right plot in Figure 2 witnesses the scan statistic exhibiting a bump around that frequency.

In the bottom panel of Figure 2 we plot averages of the statistic ${\hat{R}}_{X,0,a_{j}}$ for j=1,2,…,Q−1. We observe that this statistic is not as capable as the scan statistic ${\hat{\lambda }}_{X,a_{j}}$ in bringing out the frequency ranges of significant spectral proportions.

#### 2.1.2. Theoretical Properties of the FS-Ratio Statistic

In this section we list the required assumptions and discuss the asymptotic properties of the statistics ${\hat{r}}_{X,a,b}$ and FS-ratio ${\hat{R}}_{X,a,b}$.

**Assumption** **1.** *Let $Z_{t}={\left(X_{t},Y_{t}\right)}^{{}^{\prime }},\;t\in Z$ be a $\left(d_{1}+d_{2}\right)$-variate zero-mean second-order stationary time series. For any k>0, the kth order cumulants of $Z_{t}$ satisfy*

$$\sum_{u_{1},u_{2},\ldots ,u_{k-1}\in Z}\left[\;1+\right|u_{j}{|}^{2}\;]\;c_{b_{1},b_{2},\ldots ,b_{k}}\left(u_{1},u_{2},\ldots ,u_{k-1}\right)\;<\infty \;$$

*for j=1,2,…,k−1 and $b_{1},b_{2},\ldots ,b_{k}=1,2,\ldots ,d=d_{1}+d_{2}$ where $c_{b_{1},b_{2},\ldots ,b_{k}}\left(u_{1},u_{2},\ldots ,u_{k-1}\right)$ is the kth order joint cumulant of $Z_{b_{1},u_{1}},\ldots ,Z_{b_{k-1},u_{k-1}},Z_{b_{k},0}$ as defined in Brillinger [8].*

Please note that the *k*th order cumulant is given by $c_{b_{1},b_{2},\ldots ,b_{k}}\left(u_{1},u_{2},\ldots ,u_{k-1}\right)$$=cum\{Z_{b_{1},u_{1}},\ldots ,Z_{b_{k-1},u_{k-1}},Z_{b_{k},0}\}$ where $Z_{b_{r},u_{s}}$ refers to component $b_{r}$ of the vector $Z_{u_{s}}$ with $u_{s}$ being the time point; see Theorem 2.3.2 of Brillinger [8]. For example when k=2, the 2nd order cumulant $cum\left\{Z_{b_{1},u_{1}},Z_{b_{2},u_{2}}\right\}=cov\left(Z_{b_{1},u_{1}},Z_{b_{2},u_{2}}\right)$ is the covariance between those two random variables.

**Assumption** **2.** *(a). The kernel function K(·) is bounded, symmetric, nonnegative and Lipschitz-continuous with compact support [−π,π] and*

$$\int_{-\pi }^{\pi }K\left(\omega \right)d\omega =1.\;$$

*where K(ω) has a continuous Fourier transform k(u) such that*

$$\int k^{2}\left(u\right)du<\infty \;\;and\;\;\int k^{4}\left(u\right)du<\infty .\;$$

*(b). The bandwidth h is such that $h^{9/2}T\to 0$ and $h^{2}T\to \infty$ as T→∞.*

**Remark** **1.** *Assumptions 1 and 2 above are the same as in Eichler [9] where the first requires existence of all order moments of $Y_{t}$ and the second ensures consistency of the estimated spectral matrix. It must be noted that the assumptions on the kernel and bandwidth are primarily for establishing asymptotic result in (13) and can be weakened for Theorem 1.*

**Theorem** **1.** *Suppose that Assumptions 1,2 are satisfied. Then as T→∞,*

(8) $$\left(a\right).\;{\hat{r}}_{X,a,b}\;\overset{P}{\to }\;\int_{a}^{b}\;\sum_{r,s=1}^{d_{1}}\;f_{X,rs}\left(\omega \right)\;\bar{f_{X,rs}\left(\omega \right)}\;d\omega ,\;\;$$

*where $f_{X}\left(\omega \right)={\left(f_{X,rs}\right)}_{r,s=1,2,\ldots ,d_{1}}$ is the $d_{1}\times d_{1}$ spectral matrix of $X_{t}$ and $\overset{P}{\to }$ denotes convergence in probability. Furthermore, let ${\bar{\Pi }}_{\left(a,b\right)}=\left(0,\pi \right)\backslash \left(a,b\right)$ for some 0<a<b<π. If $r_{X,a,b}>0$ and $r_{X,{\bar{\Pi }}_{\left(a,b\right)}}>0$,*

(9) $$\left(b\right).\;{\hat{R}}_{X,a,b}\;\overset{P}{\to }\;{\left(1+\frac{r_{X,{\bar{\Pi }}_{\left(a,b\right)}}}{\;r_{X,a,b}}\right)}^{-1}\;\;$$

*where $r_{X,{\bar{\Pi }}_{\left(a,b\right)}}=\int_{{\bar{\Pi }}_{\left(a,b\right)}}\;f_{X}{\left(\omega \right)}^{2}d\omega$.*

**Proof.**  See Appendix B for details of the proof. □

Please note that in finite sample situations explored using simulation examples in Appendix A and the real data application in Section 3, we use the block bootstrap technique of Politis and Romano [10] for resampling from a stationary process. This is done to obtain sample quantiles of the FS-ratio statistic ${\hat{R}}_{X,a,b}$.

**Remark** **2.** *In a special case wherein the dimensions of the two processes are the same ($d_{1}=d_{2}$), we wish to test for the equality of spectral matrices of same dimensions over an interval 0<a<b<π. Let us assume $d_{1}=d_{2}$ and $d=d_{1}+d_{2}$ and define the d×d spectral matrix of $Z_{t}={\left(X_{t},Y_{t}\right)}^{{}^{\prime }}$ as*

(10) $$f_{Z}\left(\omega \right)\;=\;\left[\begin{array}{cc} f_{Z,11}\left(\omega \right) & f_{Z,12}\left(\omega \right)\\ f_{Z,21}\left(\omega \right) & f_{Z,22}\left(\omega \right) \end{array}\right]$$

*where the $d_{1}\times d_{1}$ matrix $f_{Z,12}\left(\omega \right)$ is the cross-spectral matrix of the processes $X_{t}$ and $Y_{t}$ and $f_{Z,11}\left(\omega \right)$ and $f_{Z,22}\left(\omega \right)$ are the spectral matrices of $X_{t}$ and $Y_{t}$ respectively. We consider testing*

(11) $$H_{0}\;:\;\;f_{X}\left(\omega \right)=f_{Y}\left(\omega \right)\;\forall \;\omega \in \left(a,b\right)$$

*where 0<a<b<π. The test statistic is*

(12) $${\hat{D}}_{X,Y}=\int_{a}^{b}||vec\left({\hat{f}}_{X}\left(\omega \right)-{\hat{f}}_{Y}\left(\omega \right)\right){\left|\right|}_{2}^{2}d\omega .$$

*The $L_{2}$ norm above in (12) on the spectral matrices is similar to the statistics considered in Eichler [9] and Dette and Paparoditis [11] wherein the problem of testing equality of spectral matrices is discussed. Suppose that Assumptions 1,2 are satisfied, an application of Theorem 3.5 of Eichler [9] yields, under $H_{0}$,*

(13) $$2\pi T\sqrt{h}\;{\hat{D}}_{X,Y}-\frac{\mu_{XY}}{\sqrt{h}}\overset{D}{\to }N\left(0,\sigma_{XY}^{2}\right)\;$$

*where*

(14) $$\mu_{XY}=A_{K}\int_{-\pi }^{\pi }1_{\omega \in (a,b)}\left(\;\sum_{p_{1},p_{2}=1}^{2}\left(\;-1\;+\;2\delta_{p_{1}p_{2}}\;\right)tr{\left(f_{Z,p_{1}p_{2}}\left(\omega \right)\right)}^{2}\right)d\omega \;$$

*and*

(15) $$\sigma_{XY}^{2}=B_{K}\int_{-\pi }^{\pi }1_{\omega \in (a,b)}\left(\;\sum_{p_{1},p_{2},p_{3},p_{4}=1}^{2}\left(\;-1\;+\;2\delta_{p_{1}p_{2}}\;\right)\;\left(\;-1\;+\;2\delta_{p_{3}p_{4}}\;\right)tr{\left(\;f_{Z,p_{1}p_{3}}^{ij}\left(\omega \right)\bar{(f_{Z,p_{2}p_{4}}^{ij}\left(\omega \right)}\right)}^{T}{\;)}^{2}\right)d\omega .\;$$

*where $\overset{D}{\to }$ denotes convergence in distribution,*

$$A_{K}=\int_{-\pi }^{\pi }K^{2}\left(v\right)dv,\;B_{K}=4\int_{a-\pi }^{b+\pi }\;{\left(\int_{-\pi }^{\pi }K\left(u\right)K\left(u+v\right)du\right)}^{2}\;dv$$

*and $\delta_{rs}=I\left(r=s\right)$ is the Kronecker delta and tr(·) denotes the trace of a matrix.*

**Remark** **3.** *In the neuroscience application in Section 3, the entire duration of the experiment is divided into non-overlapping successive time segments (a total of N epochs). Each epoch results in a multivariate stationary process of interest with the dimensions of these processes varying across epochs. Letting $X_{1,t},X_{2,t},\ldots ,X_{N,t}$ be the N stationary processes at these epochs, one can obtain the FS-ratio statistics ${\hat{R}}_{X_{i},a,b}$, for i=1,2,…,N, and view this is a series with time index being the epoch index i. Applying change point detection to this series to formally test for the significance of change points would require use of a divergence measure that measures distance between ${\hat{R}}_{X_{i},a,b}$ and ${\hat{R}}_{X_{j},a,b}$ when i≠j. Different norms can be used to construct this divergence measure and this would serve as the test statistic. Large sample distributions of this statistic would provide critical values necessary for the test. One of the issues here would be in dealing with differing errors in estimating the FS-ratio statistics when the dimensions of the two series are very different and this needs further investigation.*

## 3. Analysis of Complexity of Rat Local Field Potentials in a Stroke Experiment

In this section, we investigate the ability of the FS-ratio statistic to identify changes in the spectral properties of the local field potential (LFP) of a rat (Local field potential data on the experimental rat comes from the stroke experiment conducted at Frostig laboratory at University of California Irvine: http://frostiglab.bio.uci.edu/Home.html). The aim is to identify changes in complexity and structure of the multivariate cortex signal over the course of the experiment. It is also of interest to understand the differential roles of frequency bands and determine the specific bands that demonstrate the most significant changes that occurred due to the stroke.

At 32 locations on the rat’s cortex, microelectrodes are inserted: 4 layers in the cortex, at 300 μm, 700 μm, 1100 μm and 1500 μm and 8 microelectodes lined up in each of the 4 layers. We look at the field potential specific to the 32 locations recorded for a total duration of 10 min. This 10 min duration is divided into N=600 epochs (distinct successive non-overlapping time segments of the duration of the experiment) with each epoch comprising of 1 s worth of data. The sampling rate here is 1000 Hz resulting in T=1000 observations per epoch. Midway through the recording period (after epoch 300) a stroke is artificially induced by clamping the medial cerebral artery that supplied blood to the recorded area.

**Figure 3:**
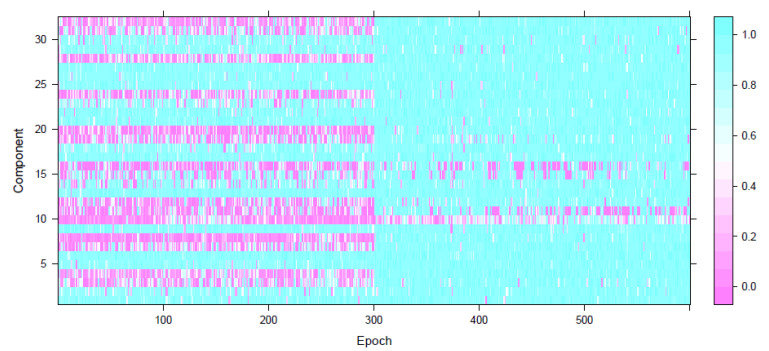
*p*-values from the test of second-order stationarity on each of the p=32 LFP microelectrodes (y-axis) for all 600 epochs (x-axis).

As a first step in our analysis, we applied a component-wise univariate test of second-order stationarity (Dwivedi and Subba Rao [12]) of the LFP signal at each epoch. In Figure 3, we present the *p*-values from a test of second-order stationarity carried out on each of the p=32 microelectrodes at each epoch. We notice that these individual microelectrodes are more stationary after the stroke than before.

Next, we model the observed 32-dimensional signal as a multivariate nonstationary time series using the stationary subspace analysis (SSA) setup. We assume the observed p=32 dimensional LFP signal $S_{i,t}$ is linearly generated by stationary and nonstationary sources in the cortex. More precisely we have,

(16) $$S_{i,t}\;=\;A_{i}X_{i,t}\;+\;\varepsilon_{i,t},\;\;i=1,2,\ldots ,N=600,$$

where $X_{i,t}\in R^{d_{i}}$ is latent stationary source, $A_{i}$ is a $p\times d_{i}$ unknown demixing matrix, $\varepsilon_{i,t}$ are the nonstationary sources. This setup of starting with an observed nonstationary time series and, after some transformation, getting to a lower dimensional stationary time series has interesting applications in neuroscience. For instance, EEG signals measuring brain activity appear often as a multivariate nonstationary time series; see Ombao et al. [13], Srinivasan [14], Nunez and Srinivasan [15], von Bünau et al. [16], Wu et al. [17], Gao et al. [18], Euán et al. [19] for examples. Kaplan et al. [20] regard the nonstationarity as background activity in the brain signal and removing this nonstationarity was seen to improve prediction accuracy in neuroscience experiments; von Bünau et al. [21] and von Bünau et al. [16]. Thus, the aim of SSA is to separate the stationary from the nonstationary sources within each epoch and focus attention on the stationary sources. From a stroke neuroscientist’s perspective, the stationary sources within a short epoch of 1 s are considered to be the “stable” components of the signal since they are consistent within that short interval. The word consistent here refers to the statistical properties of the signal remaining the same within an epoch. Of course the transient components (nonstationary components) may also be of interest in other applications.

**Figure 4:**
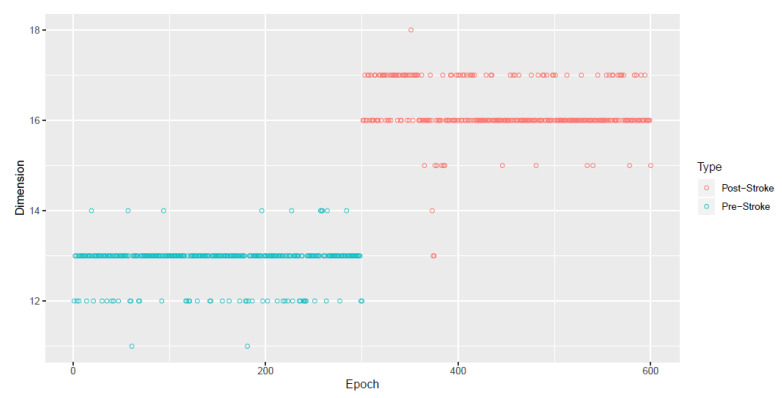
Plot of estimated stationary subspace dimensions ${\hat{d}}_{i}$ for the i=1,2,…,N=600 epochs in the stroke experiment. Please note that for each epoch *i* there is a single estimated dimension ${\hat{d}}_{i}$ that is plotted.

The next goal in the data analysis is to estimate the epoch-evolving dimension $d_{i}$ and the latent stationary time series $X_{i,t}\in R^{d_{i}}$ where $d_{i}<p$. In Figure 4, we apply SSA and plot the estimates of the stationary subspace dimension $d_{i}$ across N=600 epochs using the method in Sundararajan et al. [22].

**Figure 5:**
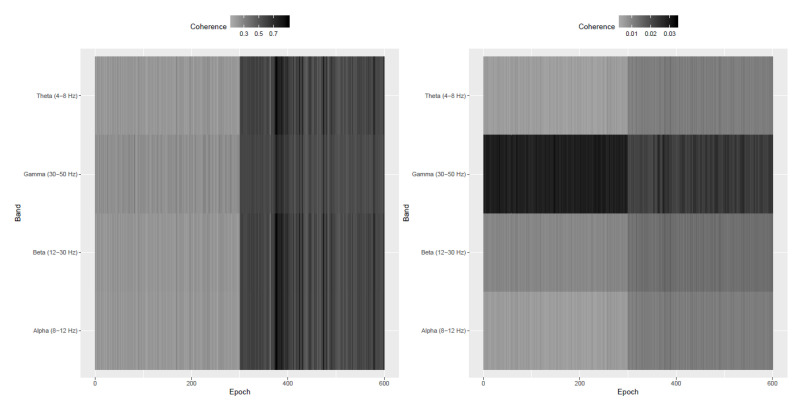
(**Left**) average squared coherence among the 32 components of the observed LFP signal across 600 epochs. The averages are computed across the specified frequency bands. (**Right**) average squared coherence among the 32 components of the pre-whitened LFP signal across 600 epochs.

The evolutionary dimension $d_{i}$ of the latent stationary sources were presented in Figure 4. The plot indicates increase in the number of stationary sources in post-stroke epochs (after epoch 300) and this agrees with the results in Figure 3 wherein more epochs after the stroke witness stationary behavior in the individual LFP components. It is indeed interesting that immediately post-occlusion (or immediately after stroke onset), the LFPs are highly synchronized: the plots of the observed LFP $S_{i,t}$ and the estimated squared coherence between the 32 components (Figure 5) suggest that different electrodes look very similar and there is high coherence in between the entire network of electrodes at various frequency bands. Please note that for the observed 32 dimensional signal $S_{i,t}$ in epoch *i*, the squared coherence between two components $S_{p,i,t}$ and $S_{q,i,t}$, for p≠q, at frequency ω is given by

(17) $$C_{p,q}\left(\omega \right)=\frac{|f_{S,pq}{\left(\omega \right)|}^{2}}{f_{S,pp}\left(\omega \right)f_{S,qq}\left(\omega \right)}$$

where $f_{S,pq}\left(\omega \right)$ denotes the cross-spectrum between those two components and $f_{S,pp}\left(\omega \right)$ and $f_{S,qq}\left(\omega \right)$ are the univariate spectra of the components series $S_{p,i,t}$ and $S_{q,i,t}$ respectively. This observation of high coherence across electrodes immediately post-occlusion was confirmed by the neuroscientists and also reported in Ellen Wann’s PhD dissertation (Wann [23]). Next, we investigate further into the lead-lag cross-dependence between microelectrodes. We pre-whitened the observed time series to make the lag-0 covariance matrix identity. More precisely, one considers $\Sigma_{i}^{-1/2}S_{i,t}$ where $\Sigma_{i}^{-1/2}$ is the inverse square root of the lag-0 covariance matrix $V(S_{i,t})$. We observe, in Figure 5, the significant drop in the magnitude of squared coherence after pre-whitening indicating that the dependence among the 32 components is predominantly due to a contemporaneous (i.e., lag-0) dependence. One can also notice, from the right plot in Figure 5, a drop in the coherence in the gamma frequency band after the stroke.

**Figure 6:**
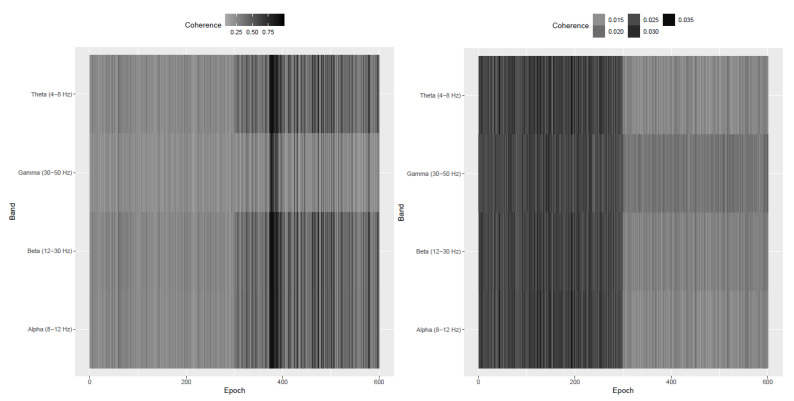
(**Left**) average squared coherence in the estimated stationary sources across 600 epochs. The averages are computed across the specified frequency bands. (**Right**) average squared coherence in the pre-whitened stationary sources across 600 epochs.

We then estimated the latent stationary sources $X_{i,t}$ for the i=1,2,…,N=600 epochs using the DSSA method in Sundararajan and Pourahmadi [24]. In order to overcome identifiability issues in the model in (16), SSA and PCA methods for time series assume an identity lag-0 covariance matrix for $X_{i,t}$ and resort to a pre-whitening technique to achieve this. Figure 6 plots the average squared coherence in the non pre-whitened and pre-whitened stationary sources across different frequency bands. Similar to the coherence pattern in the observed LFP in Figure 5, the left plot in Figure 6 witnesses an increase in the coherence after the occurrence of the stroke. This indicates the importance of the stationary components in explaining the high degree of synchronicity. Also, the right plot in Figure 6 indicates a substantial drop in the magnitude of coherence in the stationary sources. The pre-whitened stationary sources have lower coherence than the coherence of the stationary sources based on the non pre-whitened. As noted, previous findings have already indicated an increased coherence post stroke onset. Our analysis provided an additional insight that the increase in the coherence post-stroke is due only to contemporaneous (or lag-0) dependence. This indicates perfect temporal synchrony in a sense that there is no lead-lag cross-dependence between the electrodes. This was suggested by visual inspection of the LFP traces and hypothesized by neuroscientists though never formally confirmed until now with our analysis.

**Figure 7:**
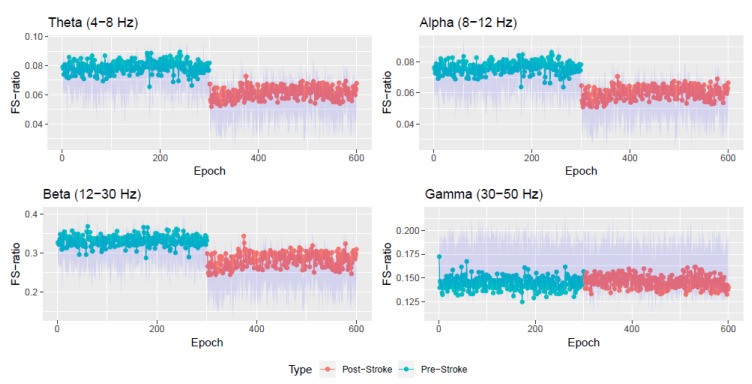
Plot of the FS-ratio statistic ${\hat{R}}_{X_{i},a,b}$ for i=1,2,…,N=600 for various frequency bands. The blue shaded region corresponds to a 95% confidence interval.

**Figure 8:**
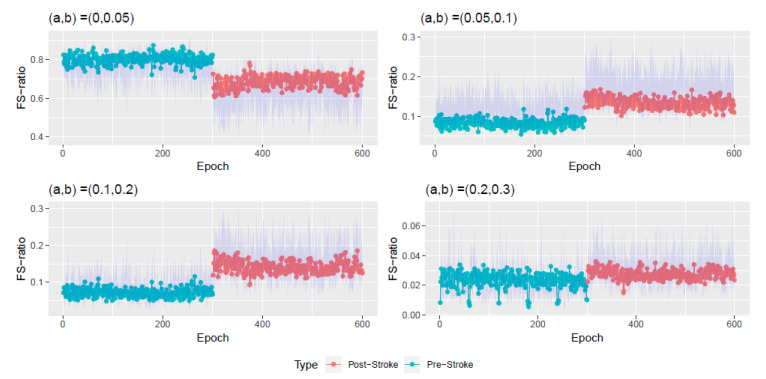
Plot of the FS-ratio statistic ${\hat{R}}_{X_{i},a,b}$ for i=1,2,…,N=600 for specified frequency ranges (a,b). Here (a,b)⊂(0,0.5) and (0,0.5) corresponds to the interval (0,π). The blue shaded region corresponds to a 95% confidence interval.

Next, the FS-ratio statistic was evaluated on these estimated stationary sources at each of the 600 epochs at various frequency bands. Figure 7 plots the estimated FS-ratio statistic ${\hat{R}}_{X_{i},a,b}$, i=1,2,…,N=600, for the known frequency bands: theta (4–8 Hertz), alpha (8–12 Hertz), beta (12–30 Hertz) and gamma (30–50 Hertz). At each epoch *i*, we obtained a 95% confidence interval for the FS-ratio statistic using the block bootstrap technique of Politis and Romano [10]. To select the block length, we follow the procedure in Politis and White [25], Patton et al. [26]. Please note that this procedure is for the univariate case and hence we apply it to each component of the multivariate process $X_{i,t}$ and obtain the block length as the average over all components. The confidence intervals are the blue shaded regions in Figure 7 and Figure 8.

The FS-ratio statistic is seen to have differences in the pre and post stroke epochs in the Theta, Alpha, and Beta bands but not in the Gamma band. It can also be seen that the biggest difference in FS-ratio between pre and post stroke is in the Beta band wherein there is a decrease in the amount of spectral information after the stroke. Figure 8 also presents the FS-ratio statistic on other specified frequency bands wherein one notices differences between the pre and post stroke epochs.

Table 1 and Table 2 contain numerical summaries of the FS-ratio statistic for the pre and post stroke epochs at various frequency bands. We notice that the Beta band is where there is maximum difference observed between the pre and post stroke epochs. The Gamma band is consistent throughout the experiment’s 600 epochs. Within the pre stroke epochs (and also within the post-stroke epochs), the most variation in FS-ratio is observed in the Beta band.

### Discussion

The *p*-values presented in Figure 3 represent a test of second-order stationarity carried out on each of the p=32 microelectrodes at each epoch. We noticed that immediately after stroke the individual microelectrodes behaved in a more stationary manner and this was visibly different from what was observed before the stroke. Based on this analysis, it might be plausible that the LFP signal, under normal circumstances, exhibits nonstationary behavior and immediately post stroke the signal behaves in a more stationary manner thereby showing that the brain’s typical functions are affected. The plots of the observed LFP $S_{i,t}$ and the estimated squared coherence between the 32 components (Figure 5) indicate high cross-electrode coherence at various frequency bands immediately post stroke. This observation was also confirmed by the neuroscientists and also reported in Ellen Wann’s PhD dissertation (Wann [23]).

In Fontaine et al. [27], a univariate LFP microelectrode-wise change point analysis was performed on the same dataset. In their work, for various frequency bands, changes in the non-linear spectral dependence of the LFP signal is modeled using parametric copulas. They detected change-points for a fixed microelectrode and fixed frequency band. One can notice the detection of numerous change points in the Delta, Theta, Alpha, Beta and Gamma bands for individual microelectrodes 1, 9 and 17. The detected change points include several epochs with very few of them being close to the time of the occlusion (or induced stroke) which was epoch i=300.

In contrast, the advantages of our method are as follows: (i). The method treats the observed LFP signal as a multivariate nonstationary time series. Using (16), we model this observed multivariate signal as a mixture of stationary and nonstationary components. Figure 4 presents the dimension of stationary subspace (dimension of $X_{i,t}$) across the 600 epochs and this is seen to be a useful feature in understanding changes in the cortical signal after the occurrence of the induced stroke (epoch 300). In other words, an increase in the dimension $d_{i}$ after the stroke points to a more stationary behavior of the LFP signal after the stroke. (ii). The FS-ratio statistic, with the ability to compare two multivariate processes with unequal dimensions, is applied on the estimated processes $X_{i,t}$ for each of the 600 epochs and frequency band specific numerical summaries are presented. The Beta frequency band is seen to be display the greatest changes within the pre stroke and post stroke epochs and also between the pre stroke and post stroke epochs. Also, from Figure 7 and Figure 8, it is very easy to spot a change point at epoch 300 when the stroke was induced.

## 4. Concluding Remarks

In this work, we proposed a new frequency-specific spectral ratio statistic FS-ratio that is demonstrated to be useful in comparing spectral information in two multivariate stationary processes of different dimensions. The method is motivated by applications in neuroscience wherein brain signal is recorded across several epochs and the widely used tactic is to assume the observed signal be linearly generated by latent sources of interest in lower dimensions. Applying PCA/ICA/SSA and other dimension reduction methods to the observed signal in different epochs in the experiment results in different estimates of the dimensions of latent sources. In these situations, the FS-ratio is seen to be useful because (i). It captures the proportion of spectral power in various frequency bands by means of a $L_{2}$-norm on the spectral matrices and (ii). It is blind to the dimension of the stationary process as it only looks at the proportion of spectral power at frequency bands. Under mild assumptions, the asymptotic properties of FS-ratio statistic are derived. We also provide a data-driven technique to locate the frequency bands that carry significant proportions of spectral power. In the application of our method to the LFP dataset, we witness the ability of our method in (i). identifying frequency bands where the pre and post stroke epochs are different, (ii). identifying frequency bands that accounts for most discrepancies within pre (and post) stroke epochs, (iii). identifying the frequency bands that are consistent across all the 600 epochs of the experiment and (iv). understanding the importance of contemporaneous dependence, both in the observed LFP and the stationary sources, across the 600 epochs and this indicated perfect synchrony (no lead-lag cross-dependence) among microelectrodes immediately after the stroke.

Topological data analysis (TDA) methods for characterizing complexity and detecting phase transitions exist in the literature; M. Piangerelli [28], Rucco et al. [29], Wang et al. [30]. Topological features from the observed series are extracted using techniques such as persistent entropy, persistence diagrams and Betti numbers and this can be viewed as another approach to identify changes in the multivariate time series due to events such as epilepsy and seizure.

## Figures and Tables

**Table 1:** Numerical summaries of FS-ratio statistic ${\hat{R}}_{X_{i},a,b}$ for **pre stroke** epochs i=1,2,…,300.

Frequency Band	Mean	Median	SD	Lower	Upper
				CI	CI
Theta (4–8 Hz)	0.079	0.079	0.004	0.061	0.081
Alpha (8–12 Hz)	0.076	0.077	0.0035	0.059	0.078
Beta (12–30 Hz)	0.332	0.332	0.0129	0.267	0.341
Gamma (30–50 Hz)	0.144	0.144	0.006	0.141	0.191

**Table 2:** Numerical summaries of FS-ratio statistic ${\hat{R}}_{X_{i},a,b}$ for **post stroke** epochs i=301,302,…,600.

Frequency Band	Mean	Median	SD	Lower	Upper
				CI	CI
Theta (4–8 Hz)	0.062	0.062	0.004	0.0422	0.0669
Alpha (8–12 Hz)	0.060	0.061	0.004	0.041	0.064
Beta (12–30 Hz)	0.283	0.285	0.018	0.202	0.292
Gamma (30–50 Hz)	0.146	0.146	0.006	0.135	0.187

**Figure A1:**
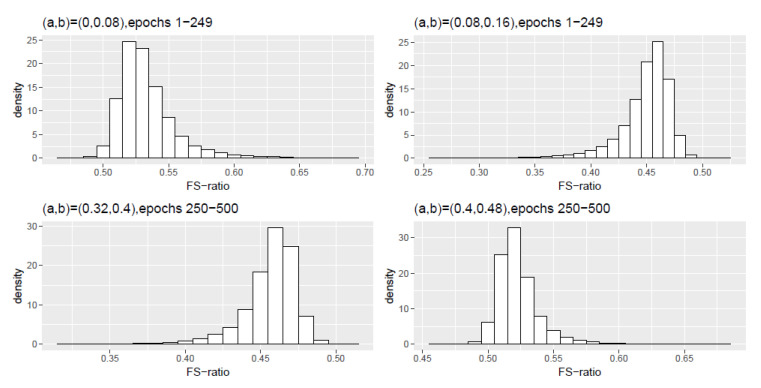
**Scheme 1**: Histogram density of the FS-ratio statistic for different frequency ranges (a,b)⊂(0,0.5).

**Figure A2:**
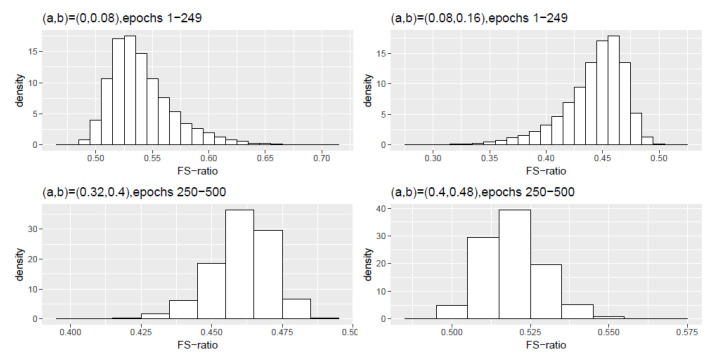
**Scheme 2**: Histogram density of the FS-ratio statistic for different frequency ranges (a,b)⊂(0,0.5).

**Figure A3:**
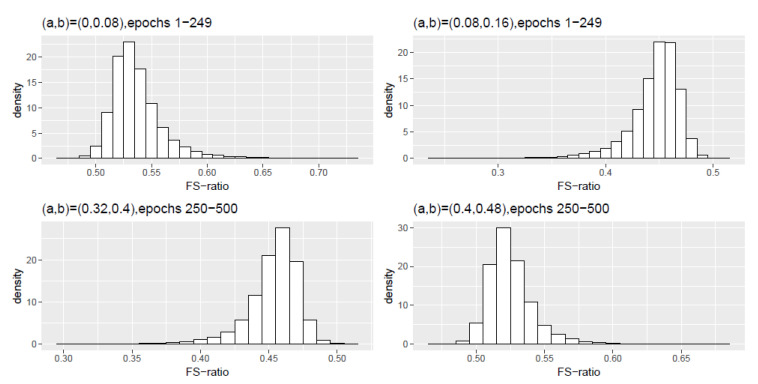
**Scheme 3**: Histogram density of the FS-ratio statistic for different frequency ranges (a,b)⊂(0,0.5).

**Figure A4:**
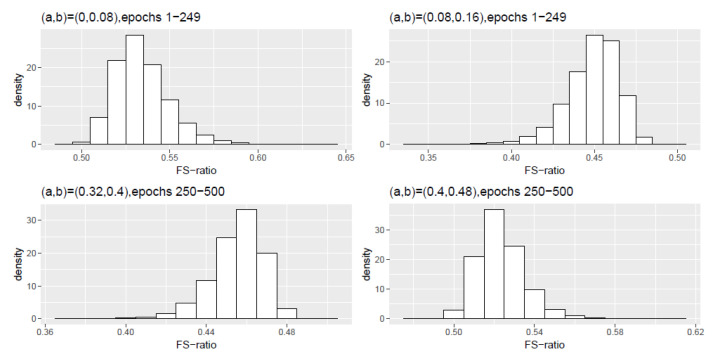
**Scheme 4**: Histogram density of the FS-ratio statistic for different frequency ranges (a,b)⊂(0,0.5).

## Appendix A. Simulation Study

In this section, we illustrate the performance of the FS-ratio statistic in capturing spread of spectral information using simulated examples. We consider four simulation schemes and report the key summaries of the FS-ratio statistic across repetitions of each of the four schemes. In addition, 95% bootstrap confidence limits for the FS-ratio statistic are computed from B=500 bootstrap replications. Here we use the block bootstrap procedure of Politis and White [25], Patton et al. [26]. For an estimate of the spectral matrix defined in (2), the Bartlett-Priestley kernel with bandwidth $h=T^{-0.3}$ and the Daniell kernel, see Example 10.4.1 in Brockwell and Davis [31] with $m=\sqrt{T}$ were implemented. Similar results were obtained for the two kernel choices and only the results from the latter are presented.

The simulation schemes presented below are designed to mimic the real data situation in Section 3. There, the entire duration of the neuroscience experiment is divided into non-overlapping successive time segments (*N* epochs). Then from these *N* epochs, lower dimensional stationary sources of varying dimensions were extracted. Similarly, in our simulations below we shall simulate *N* stationary processes with varying dimensions and investigate the evolution of the FS-ratio statistic. We now present 4 simulation schemes to assess the behavior of our FS-ratio statistic. Scheme 1 simulates *N* multivariate stationary VAR(2) processes, $X_{i,t}$ where i=1,2,…,N=500, with dimensions randomly chosen from {2,3,…,30} and the error vectors are component-wise i.i.d N(0,1). The phase parameter θ of the AR components are allowed to vary across epochs i.e two different choices, $\theta_{1}$ and $\theta_{2}$, are included with $\theta_{1}=\frac{4\pi }{25}$ for epochs i<N/2 and $\theta_{2}=\frac{4\pi }{5}$ for epochs i≥N/2. This causes a shift in the frequency bands of interest as we move across the epochs. Scheme 2 is similar to Scheme 1 but an interaction between the dimension and frequency is included. More precisely, lower dimensional signals simulated in epochs i<N/2 have a peak at frequency $\theta_{1}=\frac{4\pi }{25}$, and the higher dimensional signals simulated in epochs i≥N/2 have a peak at frequency $\theta_{2}=\frac{4\pi }{5}$. Scheme 3 is also similar to Scheme 1 but the error vectors are allowed to have contemporaneous dependence. The *N* multivariate processes in Scheme 4 are first simulated as in Scheme 1 but are then pre-multiplied with a half-orthogonal matrix. This is in line with the model assumption (16) made in Section 3.

**Scheme 1**: We simulate *N* stationary process $X_{1,t},X_{2,t},\ldots ,X_{N,t}$, for t=1,2,…,T, where the *i*th process includes a $d_{i}$-variate process $X_{i,t}={\left(X_{1,i,t},X_{2,i,t},\ldots ,X_{d_{i},i,t}\right)}^{{}^{\prime }}$ where each $X_{k,i,t}$, $k=1,2,\ldots ,d_{i}$, are independently generated univariate stationary AR(2) process given by

$$X_{k,i,t}=\phi_{i,1}X_{k,i,t-1}+\phi_{i,2}X_{k,i,t-2}+\epsilon_{k,i,t}$$

$\phi_{i,1}=2\xi_{i}\cos\left(\theta_{i}\right)$, $\phi_{i,2}=-\xi_{i}^{2}$, $\epsilon_{k,i,t}$ are i.i.d N(0,1) and $k=1,2,\ldots ,d_{i}$, i=1,2,…,N=500, t=1,2,…,T=1000. The dimension $d_{i}$ for $X_{i,t}$ is randomly chosen from {2,3,…,30}. Here $\xi_{i}∼U\left(0.8,0.98\right)$ and $\theta_{i}$ is given by

$$\theta_{i}=\left\{\begin{array}{cc} \cos(\frac{4\pi }{25}) & \mathrm{if}\;i<\frac{N}{2}\\ \cos(\frac{4\pi }{5}) & \mathrm{if}\;i\geq \frac{N}{2} \end{array}\right.$$

**Scheme 2**: We follow Scheme 1 in generating *N* process $X_{1,t},X_{2,t},\ldots ,X_{N,t}$, for t=1,2,…,T=1000 and i=1,2,…,N=500. Unlike Scheme 1, the dimension $d_{i}$ for $X_{i,t}$ is chosen such that

$$d_{i}=\left\{\begin{array}{cc} d_{1,i} & \mathrm{if}\;i<\frac{N}{2}\\ d_{2,i} & \mathrm{if}\;i\geq \frac{N}{2} \end{array}\right.$$

where $d_{1,i}$ is simulated from discrete uniform distribution over {1,2,…,14} and $d_{2,i}$ is simulated from discrete uniform distribution over {15,16,…,30}. Observe that in Scheme 2 there is an interaction between the dimension and frequency. The lower dimensional signals simulated in epochs i<N/2 has a peak at frequency $\frac{4\pi }{25}$, and the higher dimensional signals simulated in epochs i≥N/2 has a peak at frequency $\frac{4\pi }{5}$.

**Scheme 3**: Similar to Scheme 1, the $d_{i}$-variate process in the *i*th epoch $X_{i,t}={\left(X_{1,i,t},X_{2,i,t},\ldots ,X_{d_{i},i,t}\right)}^{{}^{\prime }}$ are where each $X_{k,i,t}$ are independently generated univariate stationary AR(2) process given by

$$X_{k,i,t}=\phi_{i,1}X_{k,i,t-1}+\phi_{i,2}X_{k,i,t-2}+\epsilon_{k,i,t}$$

$\phi_{i,1}=2\xi_{i}\cos\left(\theta_{i}\right)$, $\phi_{i,2}=-\xi_{i}^{2}$. The $d_{i}\times d_{i}$ variance matrix of the Gaussian noise $\epsilon_{i,t}$ is given by

$$V\left(\epsilon_{i,t}\right)=\left[\begin{array}{ccccc} 1 & \rho & \rho^{2} & \ldots & \rho^{p_{i}-1}\\ \rho & 1 & \rho & \ldots & \rho^{p_{i}-2}\\ \vdots \\ \rho^{p_{i}-1} & \rho^{p_{i}-2} & \rho^{p_{i}-3} & \ldots & 1 \end{array}\right]$$

ρ=0.4 and $k=1,2,\ldots ,d_{i}$, i=1,2,…,N=500, t=1,2,…,T=1000. The dimension $d_{i}$ for $X_{i,t}$ is randomly chosen from {2,3,…,30}. Here again, $\xi_{i}∼U\left(0.8,0.98\right)$ and $\theta_{i}$ is given by

$$\theta_{i}=\left\{\begin{array}{cc} \cos(\frac{4\pi }{25}) & \mathrm{if}i<\frac{N}{2}\\ \cos(\frac{4\pi }{5}) & \mathrm{if}i\geq \frac{N}{2} \end{array}\right.$$

**Scheme 4**: Here we let $\sum_{i=1}^{N}d_{i}=30$ and follow Scheme 1 in generating the *N* process $X_{1,t},X_{2,t},\ldots ,X_{N,T}$, for t=1,2,…,T=1000 and i=1,2,…,N=500. Then we obtain $M_{i,t}=A_{i}X_{t}$ where $A_{i}=1_{\left(i<\frac{N}{2}\right)}\;I^{d_{i}}A_{1}+1_{\left(i\geq \frac{N}{2}\right)}\;I^{d_{i}}A_{2}$ and $A_{1}$ and $A_{2}$ are two 30×30 randomly generated orthogonal matrices and $I^{d_{i}}$ is the $d_{i}\times d$ matrix containing the first $d_{i}$ rows of the identity matrix $I_{30}$. We consider $M_{i,t}\in R^{d_{i}}$ and study the spread of spectral properties across the N=500 epochs.

Table A1 and Table A2 contain the numerical summaries of the FS-ratio statistic over 100 replications of Scheme 1. Please note that the phase parameter $\theta_{i}$ for i<N/2 in Scheme 1 is at 4π/25 on a (0,π) scale or equivalently at 0.0796 on a (0,0.5) scale. We see from Table A1 that almost all of the spectral information is contained in the first two chosen frequency ranges around this peak. Similarly for i≥N/2, the phase parameter is at 4π/5 on a (0,π) scale or equivalently at 0.3981 on a (0,0.5) scale. Figure A1 plots a histogram density of the FS-ratio statistic from the 100 replications and similar histogram densities for Schemes 2, 3 and 4 can be found in Figure A2, Figure A3 and Figure A4. From Table A2 we notice that the last two chosen frequency ranges have all of the spectral information.

**Table A1:** **Scheme 1, epochs 1–249**: Numerical summaries of FS-ratio statistic ${\hat{R}}_{X_{i},a,b}$ for epochs i=1,2,…,249 for specified frequency ranges (a,b). Here (a,b)⊂(0,0.5) and (0,0.5) corresponds to the interval (0,π).

Frequency Range	Mean	Median	SD	Lower	Upper
(a,b)				CI	CI
(0,0.08)	0.5342	0.5391	0.0221	0.4984	0.6253
(0.08,0.16)	0.4486	0.4544	0.0227	0.3566	0.4831
(0.16,0.24)	0.0002	0.0002	0.0001	0.0005	0.0019
(0.24,0.32)	0	0	0	0	0.0002
(0.32,0.40)	0	0	0	0	0
(0.40,0.48)	0	0	0	0	0

**Table A2:** **Scheme 1, epochs 250–500**: Numerical summaries of FS-ratio statistic ${\hat{R}}_{X_{i},a,b}$ for epochs i=250,2,…,500 for specified frequency ranges (a,b). Here (a,b)⊂(0,0.5) and (0,0.5) corresponds to the interval (0,π).

Frequency Range	Mean	Median	SD	Lower	Upper
(a,b)				CI	CI
(0,0.08)	0	0	0	0	0
(0.08,0.16)	0	0	0	0	0
(0.16,0.24)	0	0	0	0	0
(0.24,0.32)	0.0003	0.0002	0.0002	0.0005	0.0017
(0.32,0.40)	0.4561	0.4595	0.0181	0.3786	0.4903
(0.40,0.48)	0.5205	0.5210	0.0169	0.4759	0.5826

Table A3 and Table A4 include numerical summaries of the FS-ratio statistic over 100 replications of the model in Scheme 2. Similar to Scheme 1, the phase parameter $\theta_{i}$ for i<N/2 is at 0.0796 on a (0,0.5) scale for i<N/2 and at 0.3981 on a (0,0.5) scale for i≥N/2. The results from Table A3 indicate most of the spectral information are present in the first two chosen frequency ranges. Similarly for i≥N/2, Table A4 shows that the last two chosen frequency ranges have all of the spectral information.

**Table A3:** **Scheme 2, epochs 1–249**: Numerical summaries of FS-ratio statistic ${\hat{R}}_{X_{i},a,b}$ for epochs i=1,2,…,249 for specified frequency ranges (a,b). Here (a,b)⊂(0,0.5) and (0,0.5) corresponds to the interval (0,π).

Frequency Range	Mean	Median	SD	Lower	Upper
(a,b)				CI	CI
(0,0.08)	0.5407	0.5349	0.0286	0.5041	0.6360
(0.08,0.16)	0.4423	0.4482	0.0284	0.3475	0.4778
(0.16,0.24)	0.0002	0.0003	0.0002	0.0006	0.0022
(0.24,0.32)	0	0	0	0	0.0002
(0.32,0.40)	0	0	0	0	0
(0.40,0.48)	0	0	0	0	0

**Table A4:** **Scheme 2, epochs 250–500**: Numerical summaries of FS-ratio statistic ${\hat{R}}_{X_{i},a,b}$ for epochs i=250,2,…,500 for specified frequency ranges (a,b). Here (a,b)⊂(0,0.5) and (0,0.5) corresponds to the interval (0,π).

Frequency Range	Mean	Median	SD	Lower	Upper
(a,b)				CI	CI
(0,0.08)	0	0	0	0	0
(0.08,0.16)	0	0	0	0	0
(0.16,0.24)	0	0	0	0	0.0001
(0.24,0.32)	0.0003	0.0002	0.0001	0.0005	0.0016
(0.32,0.40)	0.4605	0.4616	0.0108	0.3862	0.4938
(0.40,0.48)	0.5194	0.5186	0.0096	0.4800	0.5863

Table A5 and Table A6 contain the numerical summaries of the FS-ratio statistic over 100 replications of the model in Scheme 3. As in Scheme 1, the phase parameter $\theta_{i}$ for i<N/2 is at 0.0796 on a (0,0.5) scale for i<N/2 and at 0.3981 on a (0,0.5) scale for i≥N/2. As in Scheme 1, results from Table A5 indicate most of the spectral information are present in the first two chosen frequency ranges. Similarly for i≥N/2, Table A6 shows that the last two chosen frequency ranges have all of the spectral information.

**Table A5:** **Scheme 3, epochs 1–249**: Numerical summaries of FS-ratio statistic ${\hat{R}}_{X_{i},a,b}$ for epochs i=1,2,…,249 for specified frequency ranges (a,b). Here (a,b)⊂(0,0.5) and (0,0.5) corresponds to the interval (0,π).

Frequency Range	Mean	Median	SD	Lower	Upper
(a,b)				CI	CI
(0,0.08)	0.5371	0.5327	0.0238	0.4977	0.6284
(0.08,0.16)	0.4459	0.4504	0.0239	0.3549	0.4843
(0.16,0.24)	0.0002	0.0002	0.0001	0.0005	0.0020
(0.24,0.32)	0	0	0	0	0.0002
(0.32,0.40)	0	0	0	0	0
(0.40,0.48)	0	0	0	0	0

**Table A6:** **Scheme 3, epochs 250–500**: Numerical summaries of FS-ratio statistic ${\hat{R}}_{X_{i},a,b}$ for epochs i=250,2,…,500 for specified frequency ranges (a,b). Here (a,b)⊂(0,0.5) and (0,0.5) corresponds to the interval (0,π).

Frequency Range	Mean	Median	SD	Lower	Upper
(a,b)				CI	CI
(0,0.08)	0	0	0	0	0
(0.08,0.16)	0	0	0	0	0
(0.16,0.24)	0	0	0	0	0.0001
(0.24,0.32)	0.0003	0.0003	0.0002	0.0005	0.0018
(0.32,0.40)	0.4531	0.4566	0.0196	0.3758	0.4907
(0.40,0.48)	0.5252	0.5225	0.0172	0.4810	0.5948

Table A7 and Table A8 contain the numerical summaries of the FS-ratio statistic over 100 replications of the model in Scheme 4. Here we look at $M_{i,t}=A_{i}X_{i,t}$ which is a mixture of the components of $X_{i,t}$ generated as in Scheme 1. Please note that the peak of the spectral densities of the components of $M_{i,t}$ is still at the phase parameter $\theta_{i}$ defined in Scheme 1. Hence, the results from Table A7 and Table A8 are similar to the results from Scheme 1.

**Table A7:** **Scheme 4, epochs 1–249**: Numerical summaries of FS-ratio statistic ${\hat{R}}_{M_{i,t},a,b}$ for epochs i=1,2,…,249 for specified frequency ranges (a,b). Here (a,b)⊂(0,0.5) and (0,0.5) corresponds to the interval (0,π).

Frequency Range	Mean	Median	SD	Lower	Upper
(a,b)				CI	CI
(0,0.08)	0.5342	0.5321	0.0155	0.4871	0.5955
(0.08,0.16)	0.4489	0.4510	0.0159	0.3872	0.4949
(0.16,0.24)	0.0002	0.0002	0.0001	0.0003	0.0011
(0.24,0.32)	0.0001	0.0001	0	0	0.0001
(0.32,0.40)	0	0	0	0	0
(0.40,0.48)	0	0	0	0	0

**Table A8:** **Scheme 4, epochs 250–500**: Numerical summaries of FS-ratio statistic ${\hat{R}}_{M_{i,t},a,b}$ for epochs i=250,2,…,500 for specified frequency ranges (a,b). Here (a,b)⊂(0,0.5) and (0,0.5) corresponds to the interval (0,π).

Frequency Range	Mean	Median	SD	Lower	Upper
(a,b)				CI	CI
(0,0.08)	0	0	0	0	0
(0.08,0.16)	0	0	0	0	0
(0.16,0.24)	0	0	0	0	0.0001
(0.24,0.32)	0.0003	0.0003	0.0001	0.0003	0.0011
(0.32,0.40)	0.4553	0.4570	0.0130	0.4021	0.4987
(0.40,0.48)	0.5234	0.5219	0.0119	0.4782	0.5738

## Appendix B. Proofs

Here we present the proofs of the theoretical results in Section 2.1.2.

**Proof of Theorem** **1** **(a).** Recall that for some 0<a<b<π,

$$\begin{array}{c} {\hat{r}}_{X,a,b}=\int_{a}^{b}||vec\left({\hat{f}}_{X}\left(\omega \right)\right){\left|\right|}_{2}^{2}d\omega =\int_{a}^{b}\left|\right|\frac{1}{T}\sum_{j=-\lfloor T/2\rfloor }^{\left\lfloor T/2\right\rfloor }\;K_{h}\left(\omega -\omega_{j}\right)vec\left(I_{X,T}\left(\omega_{j}\right)\right){\left|\right|}^{2}\;d\omega \\ =\int_{a}^{b}\frac{1}{T^{2}}\sum_{j_{1},j_{2}=-\left\lfloor T/2\right\rfloor }^{\left\lfloor T/2\right\rfloor }\;K_{h}\left(\omega -\omega_{j_{1}}\right)K_{h}\left(\omega -\omega_{j_{2}}\right)\sum_{r,s=1}^{d_{i}}\;I_{X,T,rs}\left(\omega_{j_{1}}\right)\bar{I_{X,T,rs}\left(\omega_{j_{2}}\right)}\;d\omega . \end{array}$$

We first consider the expected value of this quantity.

$$\begin{array}{c} E\left({\hat{r}}_{X,a,b}\right)=\int_{a}^{b}\frac{1}{T^{2}}\sum_{j_{1},j_{2}=-\left\lfloor T/2\right\rfloor }^{\left\lfloor T/2\right\rfloor }\;K_{h}\left(\omega -\omega_{j_{1}}\right)K_{h}\left(\omega -\omega_{j_{2}}\right)\sum_{r,s=1}^{d_{i}}\;E\left(I_{X,T,rs}\left(\omega_{j_{1}}\right)\bar{I_{X,T,rs}\left(\omega_{j_{2}}\right)}\right)\;d\omega \\ =\int_{a}^{b}\frac{1}{T^{2}}\sum_{j_{1},j_{2}=-\left\lfloor T/2\right\rfloor }^{\left\lfloor T/2\right\rfloor }\;K_{h}\left(\omega -\omega_{j_{1}}\right)K_{h}\left(\omega -\omega_{j_{2}}\right)\sum_{r,s=1}^{d_{i}}\;f_{X,rs}\left(\omega_{j_{1}}\right)\bar{f_{X,rs}\left(\omega_{j_{2}}\right)}\;d\omega \;+\;o\left(1\right). \end{array}$$

It can be seen that as T→∞, h→0 and Th→∞ the above quantity converges to

$$\begin{array}{c} \int_{a}^{b}\;\sum_{r,s=1}^{d_{i}}{\left(\int_{-\pi }^{\pi }K\left(v\right)dv\right)}^{2}\;f_{X,rs}\left(\omega \right)\;\bar{f_{X,rs}\left(\omega \right)}\;d\omega \;=\;\int_{a}^{b}\;\sum_{r,s=1}^{d_{i}}\;f_{X,rs}\left(\omega \right)\;\bar{f_{X,rs}\left(\omega \right)}\;d\omega . \end{array}$$

Next, for the variance we have $V\left({\hat{r}}_{X,a,b}\right)=A_{1}-A_{2}$, where

$$\begin{array}{c} A_{1}=\int_{a}^{b}\int_{a}^{b}\frac{1}{T^{4}}\sum_{j_{1},j_{2},j_{3},j_{4}}K_{h}\left(\omega -\omega_{j_{1}}\right)K_{h}\left(\omega -\omega_{j_{2}}\right)K_{h}\left(\lambda -\omega_{j_{3}}\right)K_{h}\left(\lambda -\omega_{j_{4}}\right)\;\times \\ \sum_{r,s,t,u=1}^{d_{i}}E\left(I_{X,T,rs}\left(\omega_{j_{1}}\right)\bar{I_{X,T,rs}\left(\omega_{j_{2}}\right)}I_{X,T,tu}\left(\omega_{j_{3}}\right)\bar{I_{X,T,tu}\left(\omega_{j_{4}}\right)}\right)\;d\omega \;d\lambda \;\;\mathrm{and}\\ A_{2}=\int_{a}^{b}\int_{a}^{b}\frac{1}{T^{4}}\sum_{j_{1},j_{2},j_{3},j_{4}}K_{h}\left(\omega -\omega_{j_{1}}\right)K_{h}\left(\omega -\omega_{j_{2}}\right)K_{h}\left(\lambda -\omega_{j_{3}}\right)K_{h}\left(\lambda -\omega_{j_{4}}\right)\;\times \\ \sum_{r,s,t,u=1}^{d_{i}}E\left(I_{X,T,rs}\left(\omega_{j_{1}}\right)\bar{I_{X,T,rs}\left(\omega_{j_{2}}\right)}\right)E\left(I_{X,T,tu}\left(\omega_{j_{3}}\right)\bar{I_{X,T,tu}\left(\omega_{j_{4}}\right)}\right)\;d\omega \;d\lambda . \end{array}$$

For the difference in the expectations between $A_{1}$ and $A_{2}$ we discuss the relevant cases and their convergence to 0. Firstly, it can be seen that for the following three cases the difference in the expectations is asymptotically 0: (a). $\omega_{j_{1}}\neq \omega_{j_{2}}\neq \omega_{j_{3}}\neq \omega_{j_{4}}$, (b). $\omega_{j_{1}}=\omega_{j_{2}}\neq \omega_{j_{3}}\neq \omega_{j_{4}}$, (c). $\omega_{j_{1}}=\omega_{j_{2}}\neq \omega_{j_{3}}=\omega_{j_{4}}$. Next, when $\omega_{j_{1}}=\omega_{j_{3}}\neq \omega_{j_{2}}=\omega_{j_{4}}$ we have,

$$\begin{array}{c} \int_{a}^{b}\int_{a}^{b}\frac{1}{T^{4}}\sum_{j_{1},j_{2}}K_{h}\left(\omega -\omega_{j_{1}}\right)K_{h}\left(\omega -\omega_{j_{2}}\right)K_{h}\left(\lambda -\omega_{j_{1}}\right)K_{h}\left(\lambda -\omega_{j_{2}}\right)\sum_{r,s,t,u=1}^{d_{i}}[E(I_{X,T,rs}\left(\omega_{j_{1}}\right)\bar{I_{X,T,rs}\left(\omega_{j_{2}}\right)}\times \\ I_{X,T,tu}\left(\omega_{j_{1}}\right)\bar{I_{X,T,tu}\left(\omega_{j_{2}}\right)})-E\left(I_{X,T,rs}\left(\omega_{j_{1}}\right)\bar{I_{X,T,rs}\left(\omega_{j_{2}}\right)}\right)E\left(I_{X,T,tu}\left(\omega_{j_{1}}\right)\bar{I_{X,T,tu}\left(\omega_{j_{2}}\right)}\right)]d\omega \;d\lambda \\ =\;\int_{a}^{b}\int_{a}^{b}\frac{1}{T^{4}}\sum_{j_{1},j_{2}}K_{h}\left(\omega -\omega_{j_{1}}\right)K_{h}\left(\omega -\omega_{j_{2}}\right)K_{h}\left(\lambda -\omega_{j_{1}}\right)K_{h}\left(\lambda -\omega_{j_{2}}\right)\sum_{r,s,t,u=1}^{d_{i}}[E\left(I_{X,T,rs}\left(\omega_{j_{1}}\right)I_{X,T,tu}\left(\omega_{j_{1}}\right)\right)\times \\ E\left(\bar{I_{X,T,rs}\left(\omega_{j_{2}}\right)}\bar{I_{X,T,tu}\left(\omega_{j_{2}}\right)}\right)-E\left(I_{X,T,rs}\left(\omega_{j_{1}}\right)\right)E\left(\bar{I_{X,T,rs}\left(\omega_{j_{2}}\right)}\right)E\left(I_{X,T,tu}\left(\omega_{j_{1}}\right)\right)E\left(\bar{I_{X,T,tu}\left(\omega_{j_{2}}\right)}\right)]d\omega \;d\lambda +o\left(1\right)\\ =\;\frac{1}{T^{4}}\sum_{j_{1},j_{2}}{\left(\int_{a}^{b}K_{h}\left(\omega -\omega_{j_{1}}\right)K_{h}\left(\omega -\omega_{j_{2}}\right)\;d\omega \right)}^{2}\;\sum_{r,s,t,u=1}^{d_{i}}[\left(f_{X,rt}\left(\omega_{j_{1}}\right)f_{X,su}\left(\omega_{j_{1}}\right)+f_{X,rs}\left(\omega_{j_{1}}\right)f_{X,tu}\left(\omega_{j_{1}}\right)\right)\times \\ \left(\bar{f_{X,rt}\left(\omega_{j_{2}}\right)}\;\bar{f_{X,su}\left(\omega_{j_{2}}\right)}+\bar{f_{X,rs}\left(\omega_{j_{2}}\right)}\;\bar{f_{X,tu}\left(\omega_{j_{2}}\right)}\right)-\left(f_{X,rs}\left(\omega_{j_{1}}\right)\bar{f_{X,rs}\left(\omega_{j_{2}}\right)}f_{X,tu}\left(\omega_{j_{1}}\right)\bar{f_{X,tu}\left(\omega_{j_{2}}\right)}\right)]\\ \;+\;o\left(1\right)\;=\frac{1}{T^{4}h^{2}}\sum_{j_{1},j_{2}}{\left(\int_{\frac{a-\omega_{j_{1}}}{h}}^{\frac{b-\omega_{j_{1}}}{h}}K\left(u\right)K\left(u+\frac{\omega_{j_{1}}-\omega_{j_{2}}}{h}\right)du\right)}^{2}\;\sum_{r,s,t,u=1}^{d_{i}}\left[\cdots \right]\;=\;O\left(\frac{1}{T^{2}h}\right). \end{array}$$

The case when $\omega_{j_{1}}=\omega_{j_{2}}=\omega_{j_{3}}\neq \omega_{j_{4}}$ would have the same rate of decay as above. Next, when $\omega_{j_{1}}=\omega_{j_{3}}\neq \omega_{j_{2}}\neq \omega_{j_{4}}$ we have,

$$\begin{array}{c} \int_{a}^{b}\int_{a}^{b}\frac{1}{T^{4}}\sum_{j_{1},j_{2},j_{4}}K_{h}\left(\omega -\omega_{j_{1}}\right)K_{h}\left(\omega -\omega_{j_{2}}\right)K_{h}\left(\lambda -\omega_{j_{1}}\right)K_{h}\left(\lambda -\omega_{j_{4}}\right)\sum_{r,s,t,u=1}^{d_{i}}[E(I_{X,T,rs}\left(\omega_{j_{1}}\right)\bar{I_{X,T,rs}\left(\omega_{j_{2}}\right)}\times \\ I_{X,T,tu}\left(\omega_{j_{1}}\right)\bar{I_{X,T,tu}\left(\omega_{j_{4}}\right)})-E\left(I_{X,T,rs}\left(\omega_{j_{1}}\right)\bar{I_{X,T,rs}\left(\omega_{j_{2}}\right)}\right)E\left(I_{X,T,tu}\left(\omega_{j_{1}}\right)\bar{I_{X,T,tu}\left(\omega_{j_{4}}\right)}\right)]d\omega \;d\lambda \\ =\;\int_{a}^{b}\int_{a}^{b}\frac{1}{T^{4}}\sum_{j_{1},j_{2},j_{4}}K_{h}\left(\omega -\omega_{j_{1}}\right)K_{h}\left(\omega -\omega_{j_{2}}\right)K_{h}\left(\lambda -\omega_{j_{1}}\right)K_{h}\left(\lambda -\omega_{j_{4}}\right)\sum_{r,s,t,u=1}^{d_{i}}[E\left(I_{X,T,rs}\left(\omega_{j_{1}}\right)I_{X,T,tu}\left(\omega_{j_{1}}\right)\right)\times \\ E\left(\bar{I_{X,T,rs}\left(\omega_{j_{2}}\right)}\right)E\left(\bar{I_{X,T,tu}\left(\omega_{j_{4}}\right)}\right)-E\left(I_{X,T,rs}\left(\omega_{j_{1}}\right)\right)E\left(\bar{I_{X,T,rs}\left(\omega_{j_{2}}\right)}\right)E\left(I_{X,T,tu}\left(\omega_{j_{1}}\right)\right)E\left(\bar{I_{X,T,tu}\left(\omega_{j_{4}}\right)}\right)]d\omega \;d\lambda +o\left(1\right)\\ =\;\frac{1}{T^{4}}\sum_{j_{1},j_{2},j_{4}}\left(\int_{a}^{b}K_{h}\left(\omega -\omega_{j_{1}}\right)K_{h}\left(\omega -\omega_{j_{2}}\right)\;d\omega \right)\left(\int_{a}^{b}K_{h}\left(\lambda -\omega_{j_{1}}\right)K_{h}\left(\lambda -\omega_{j_{4}}\right)\;d\lambda \right)\sum_{r,s,t,u=1}^{d_{i}}[(f_{X,rt}\left(\omega_{j_{1}}\right)\times \\ f_{X,su}\left(\omega_{j_{1}}\right)+f_{X,rs}\left(\omega_{j_{1}}\right)f_{X,tu}\left(\omega_{j_{1}}\right))\times \left(\bar{f_{X,rs}\left(\omega_{j_{2}}\right)}\;\bar{f_{X,tu}\left(\omega_{j_{4}}\right)}\right)-\left(f_{X,rs}\left(\omega_{j_{1}}\right)\bar{f_{X,rs}\left(\omega_{j_{2}}\right)}f_{X,tu}\left(\omega_{j_{1}}\right)\bar{f_{X,tu}\left(\omega_{j_{4}}\right)}\right)]\\ \;+\;o\left(1\right)=\frac{1}{T^{4}h^{2}}\sum_{j_{1},j_{2},j_{4}}\left(\int_{\frac{a-\omega_{j_{1}}}{h}}^{\frac{b-\omega_{j_{1}}}{h}}K\left(u\right)K\left(u+\frac{\omega_{j_{1}}-\omega_{j_{2}}}{h}\right)\;du\right)\left(\int_{\frac{a-\omega_{j_{1}}}{h}}^{\frac{b-\omega_{j_{1}}}{h}}K\left(v\right)K\left(v+\frac{\omega_{j_{1}}-\omega_{j_{4}}}{h}\right)\;dv\right)\times \\ \sum_{r,s,t,u=1}^{d_{i}}\;\left[\;\cdots \;\right]\;+o\left(1\right)\;=\;O\left(\frac{1}{T}\right). \end{array}$$

Finally, we look at the case $\omega_{j_{1}}=\omega_{j_{2}}=\omega_{j_{3}}=\omega_{j_{4}}$. We have

$$\begin{array}{c} \int_{a}^{b}\int_{a}^{b}\frac{1}{T^{4}}\sum_{j_{1}}K_{h}^{2}\left(\omega -\omega_{j_{1}}\right)K_{h}^{2}\left(\lambda -\omega_{j_{1}}\right)\sum_{r,s,t,u=1}^{d_{i}}[E(I_{X,T,rs}\left(\omega_{j_{1}}\right)\bar{I_{X,T,rs}\left(\omega_{j_{1}}\right)}\times \\ I_{X,T,tu}\left(\omega_{j_{1}}\right)\bar{I_{X,T,tu}\left(\omega_{j_{1}}\right)})-E\left(I_{X,T,rs}\left(\omega_{j_{1}}\right)\bar{I_{X,T,rs}\left(\omega_{j_{1}}\right)}\right)E\left(I_{X,T,tu}\left(\omega_{j_{1}}\right)\bar{I_{X,T,tu}\left(\omega_{j_{1}}\right)}\right)]d\omega \;d\lambda \\ =\frac{1}{T^{4}}\sum_{j_{1}}{\left(\int_{a}^{b}K_{h}^{2}\left(\omega -\omega_{j_{1}}\right)\;d\omega \right)}^{2}\sum_{r,s,t,u=1}^{d_{i}}\left[\cdots \right]=\frac{1}{T^{4}h^{4}}\sum_{j_{1}}{\left(\int_{a}^{b}K^{2}\left(\frac{\omega -\omega_{j_{1}}}{h}\right)d\omega \right)}^{2}\sum_{r,s,t,u=1}^{d_{i}}\left[\cdots \right]\\ =\frac{1}{T^{4}h^{2}}\sum_{j_{1}}{\left(\int_{\frac{a-\omega_{j_{1}}}{h}}^{\frac{b-\omega_{j_{1}}}{h}}K^{2}\left(u\right)du\right)}^{2}\sum_{r,s,t,u=1}^{d_{i}}\left[\cdots \right]=\;O\left(\frac{1}{T^{3}h^{2}}\right) \end{array}$$

□

**Proof of Theorem** **1** **(b).** First, we observe that

(A1) $$\begin{array}{c} {\hat{R}}_{X,a,b}=\frac{\int_{a}^{b}||vec\left({\hat{f}}_{X}\left(\omega \right)\right){\left|\right|}_{2}^{2}d\omega }{\int_{0}^{\pi }||vec\left({\hat{f}}_{X}\left(\omega \right)\right){\left|\right|}_{2}^{2}d\omega }=\frac{\int_{a}^{b}||vec\left({\hat{f}}_{X}\left(\omega \right)\right){\left|\right|}_{2}^{2}d\omega }{\int_{a}^{b}||vec\left({\hat{f}}_{X}\left(\omega \right)\right){\left|\right|}_{2}^{2}d\omega +\int_{{\bar{\Pi }}_{\left(a,b\right)}}||vec\left({\hat{f}}_{X}\left(\omega \right)\right){\left|\right|}_{2}^{2}d\omega }\\ ={\left(1+\frac{\int_{{\bar{\Pi }}_{\left(a,b\right)}}||vec\left({\hat{f}}_{X}\left(\omega \right)\right){\left|\right|}_{2}^{2}d\omega }{\int_{a}^{b}||vec\left({\hat{f}}_{X}\left(\omega \right)\right){\left|\right|}_{2}^{2}d\omega }\right)}^{-1}. \end{array}$$

A sufficient condition for joint consistency of ${\left({\hat{r}}_{X,a,b},{\hat{r}}_{X,{\bar{\Pi }}_{\left(a,b\right)}}\right)}^{⊤}$. Following the proof of Theorem 1 (a), we have $cov\left({\hat{r}}_{X,a,b},{\hat{r}}_{X,{\bar{\Pi }}_{\left(a,b\right)}}\right)=C_{1}-C_{2}$, where

$$\begin{array}{c} C_{1}=\int_{{\bar{\Pi }}_{\left(a,b\right)}}\int_{a}^{b}\frac{1}{T^{4}}\sum_{j_{1},j_{2},j_{3},j_{4}}K_{h}\left(\omega -\omega_{j_{1}}\right)K_{h}\left(\omega -\omega_{j_{2}}\right)K_{h}\left(\lambda -\omega_{j_{3}}\right)K_{h}\left(\lambda -\omega_{j_{4}}\right)\;\times \\ \sum_{r,s,t,u=1}^{d_{i}}E\left(I_{X,T,rs}\left(\omega_{j_{1}}\right)\bar{I_{X,T,rs}\left(\omega_{j_{2}}\right)}I_{X,T,tu}\left(\omega_{j_{3}}\right)\bar{I_{X,T,tu}\left(\omega_{j_{4}}\right)}\right)\;d\omega \;d\lambda \;\;\mathrm{and}\\ C_{2}=\int_{{\bar{\Pi }}_{\left(a,b\right)}}\int_{a}^{b}\frac{1}{T^{4}}\sum_{j_{1},j_{2},j_{3},j_{4}}K_{h}\left(\omega -\omega_{j_{1}}\right)K_{h}\left(\omega -\omega_{j_{2}}\right)K_{h}\left(\lambda -\omega_{j_{3}}\right)K_{h}\left(\lambda -\omega_{j_{4}}\right)\;\times \\ \sum_{r,s,t,u=1}^{d_{i}}E\left(I_{X,T,rs}\left(\omega_{j_{1}}\right)\bar{I_{X,T,rs}\left(\omega_{j_{2}}\right)}\right)E\left(I_{X,T,tu}\left(\omega_{j_{3}}\right)\bar{I_{X,T,tu}\left(\omega_{j_{4}}\right)}\right)\;d\omega \;d\lambda . \end{array}$$

As in the proof of Theorem 1 (a), it can be seen that, for the various cases, the covariance terms are of $O(\frac{1}{T^{\delta_{1}}h^{\delta_{2}}})$ where $\delta_{1},\delta_{2}\in \left\{0,1,2,3\right\}$ and $\delta_{1}>\delta_{2}$. The result above along with Theorem 1 implies

$${\left({\hat{r}}_{X,a,b},{\hat{r}}_{X,{\bar{\Pi }}_{\left(a,b\right)}}\right)}^{⊤}\;\overset{P}{\to }\;{\left(r_{X,a,b},r_{X,{\bar{\Pi }}_{\left(a,b\right)}}\right)}^{⊤}.$$

Finally, an application of the continuous mapping theorem yields the result. □
